# Supplementary material for: Scalable Reporter Assays to Analyze the Regulation of stx2 Expression in Shiga Toxin-Producing Enteropathogens
Source: Toxins (Basel). 2021 Jul 29;13(8):534. doi: 10.3390/toxins13080534 (PMC8402550; doi:10.3390/toxins13080534)
Supplement: Supplementary file 1 [file toxins-13-00534-s001.zip › toxins-1274411-supplementary doi added.pdf]

Supplementary Materials: Scalable Reporter Assays to Analyze the Regulation of *stx2* Expression in Shiga Toxin-Producing Enteropathogens

Martin B. Koeppel, Jana Glaser, Tobias Baumgartner, Stefanie Spriewald, Roman G. Gerlach, Benedikt v. Armansperg, John M. Leong and Bärbel Stecher

Table S1. Information for Reporter strain construction.

Strain	Template Plasmid	Forward Primer	Reverse Primer	Proof Primer Fw	Proof Primer Rev
MBK1	pWRG7	Stx2-SFGFP fusion Fw	Stx2_Com fusion Rev	Stx2 operon Fw	Stx2 operon Rev
MBK4	pWRG7	Stx2-SFGFP fusion Fw	Stx2 R/S Com fusion Rev	Stx2 operonFw	R/S outside Rev
MBK6	P3121	Stx2-Luc fusion Fw	Stx2_Com fusion Rev	Stx2 operonFw	Stx2 operon Rev
MBK7	P3121	Stx2-Luc fusion Fw	Stx2 R/S Com fusion Rev	Stx2 operon Fw	R/S outside Rev
MBK22	pMBK3	not applicable (linearized plasmid was used as template DNA)		Stx2A outside Fw	DBS770 outside Rev
JLG6	pWRG701	Stx2-Gluc fusion Fw	Stx2 R/SCom fusion Rev	Stx2A outside Fw	R/S outside Rev
JLG11	pJLG1	Stx2-T7 pol fusion	Stx2_Com fusion Rev	Stx2A outside Fw	Stx2B primer Rev
JLG12	pJLG1	Stx2-T7 pol fusion	Stx2 R/S Com fusion Rev	Stx2A outside Fw	R/S outside Rev

Table S2. Primers used in this study.

Designation	Sequence (5′-3′)	Reference
Stx2 operon Fw	GATCGGTATGTTGAGCGTGA	This study
Stx2 operon Rev	TGCTCAGTCTGACAGGCAAC	This study
Stx2-SFGFP fusion Fw	TATATCTGCGCCGGGTCTGGTGCTGAT-TACTTCAGCCAAAAGGAACACCTGTATATGCG-CAAAGGCGAAGAACTGTTTAC	This study
Stx2_Com fusion Rev	ATTAACAGAAGCTAATGCAAATAAAACCGCCA-TAAACATCTTCTTCATGCTTAACTCCTCGTGTAGGCTGGAGCTGCTTC	[35]
Stx2 R/S Com fusion Rev	CAGGATTTCGTGTTATCCGTCCAGGTAAGCAAACCTCATTTTTCAG-CAAAATATTCTTCCGTGTAGGCTGGAGCTGCTTC	This study
Stx2-Luc fusion Fw	TTATATCTGCGCCGGGTCTGGTGCTGAT-TACTTCAGCCAAAAGGAACACCTGTATATGGAA-GACGCCAAAAACATAAGAA	This study
Stx2-Gluc fusion Fw	TTTTATATCTGCGCCGGGTCTGGTGCTGAT-TACTTCAGCCAAAAGGAACACCTG-TATATGAAACCGACCGAAAACAACGA	[35]
Stx2-T7 pol fusion	TATATCTGCGCCGGGTCTGGTGCTGAT-TACTTCAGCCAAAAGGAACACCTGTATATGAACAC-GATTAACATCGCTAAGAA	This study
Stx2A outside Fw	AGACGGTCAGGGAAGTTCAG	[35]
Stx2B primer Rev	AATCCGGAGCCTGATTCACA	[35]
R/S outside Rev	GAGATGCGCAGAAATGACAA	This study
DBS770 outside Rev	GCGCGGATCCTTCGAATAAA	[35]
GA DBS Stx2 up Fw	GGCTGACATGGGAATTCCTGCAGCCCGGGGGATCCTCAGTCAGAAC-GGATGATATTGCAG	[35]
GA DBS Stx2 up Rev	TTCGGTCGGTTTCATATACAGGTGTTCCTTTTGGCTGAAG	[35]
GA DBS GlucKan Fw	AAGGAACACCTGTATATGAAACCGACCGAAAACAACGAAG	[35]
GA DBS GlucKan Rev	AGCTAAGGAAGCTAACGTGTAGGCTGGAGCTGC	[35]

GA DBS Cm dn Fw	GCTCCAGCCTACACGTTAGCTTCCTTAGCTCCTGAAAATCTC	This study
GA DBS Cm dn Rev	AAGCTCAATAAAAAGCCCCACCGCGGTGGCGGCCGCTTGATCCG-CAGGCGCTTAC	This study
